# Treatment of Disturbed Sleep in Progressive Supranuclear Palsy: a randomized, remote, double-blinded, 6-week cross-over design study protocol comparing zolpidem, suvorexant, and placebo

**DOI:** 10.1186/s13063-025-09382-9

**Published:** 2026-01-07

**Authors:** Esther Li, Felicia Song, Quentin Coppola, Leslie Yack, Michael Le, Samirah Javed, Natalie Pandher, Igor Prufer, Olga Mayzel, Hilary H. Heuer, Mary Koestler, Bruce L. Miller, Adam L. Boxer, Lawren Vandevrede, Lea T. Grinberg, Christine M. Walsh, Thomas C. Neylan

**Affiliations:** 1 University of California, San Francisco, San Francisco, USA; 2 Department of Psychiatry, UCSF, San Francisco, CA USA; 3 Department of Psychiatry, UCSF, School of Medicine UCSD, San Francisco, CA USA; 4 Department of Psychology, Northeastern University, Clinical Research Coordinator, Psychiatry UCSF, San Francisco, CA USA; 5 Department of Psychiatry, UCSF, SFVA, San Francisco, CA USA; 6 Department of Psychiatry, UCSF, California Health Sciences University, San Francisco, CA USA; 7 Department of Psychiatry, UCSF, Google Deep Mind Program, San Francisco, CA USA; 8 Department of Epidemiology, UCD, San Francisco, CA USA; 9 Global Brain Health Initiative, UCSF, San Francisco, CA USA; 10 Department of Neurology, UCSF, San Francisco, CA USA; 11 Department of Neurology, Mayo Clinic Jacksonville, San Francisco, CA USA; 12 Departments of Neurology & Psychiatry, UCSF; SFVA, San Francisco, CA USA

**Keywords:** Remote, Clinical trial, FDA-approved, Hypnotics, Sleep, Progressive supranuclear palsy, Neurodegenerative disease, Virtual, Social engagement platform, Telemedicine, Burden reduction, Decentralized trial, Mobile trial

## Abstract

**Background:**

Prior research identified profound sleep disruption in progressive supranuclear palsy (PSP). The hypothalamus and brainstem, areas that help regulate sleep/wake patterns, are among the earliest affected brain regions in PSP disease progression. Comparing polysomnography and quantitative-neuropathology metrics, we identified relative sparing of wake-promoting nuclei in PSP compared to Alzheimer’s disease, though PSP had more disrupted sleep. It led to the hypothesis that PSP patients have hyperinsomnia (or hyposomnia, little sleep) due to degeneration of sleep nuclei with a preservation of sleep neurons, causing a system unbalance. A higher neuronal count of wake-promoting nuclei was associated with greater nocturnal wake, regardless of disease. Specifically, orexinergic wake-promoting neurons in the lateral hypothalamus, previously described as the sleep-on/off switch, are relatively spared in PSP. Thus, we hypothesized that an orexinergic antagonist may be more effective in treating sleep/wake issues in PSP than other hypnotic medications. This study protocol was established to test the safety and efficacy of an orexinergic antagonist (suvorexant) targeting the wake-promoting system and contrasts it with a GABAergic receptor agonist (zolpidem) targeting sleep-promoting systems and placebo.

**Methods:**

This is a remote clinical trial, designed as a double-blind, cross-over, within-subject 6-week trial, with 3 one-week-long conditions, separated by 1-week washout periods. The order of the 3 regimens is randomized and counterbalanced: placebo (microcrystalline cellulose), 15 mg/day suvorexant, 5 mg/day zolpidem. Participants are recruited from doctor and study referrals, registries, and support groups. Once onboarded, the trial coordinator maintains communication with the participant/caregiver throughout the 6 weeks. Assessments include neurological interviews, cognitive testing, and subjective questionnaire packets. Sleep and circadian rhythms are assessed through ambulatory EEG and actigraphy monitoring devices worn by the participant throughout the trial.

**Discussion:**

The study design aims to reduce participant and caregiver burden, while improving accessibility to such a study. Administering a remote clinical trial for a rare disease, however, creates unique issues that would otherwise be absent from in-person studies. Particularly, a symptom rather than disease-modifying trial is challenging to recruit for when potential disease-modifying therapeutics are available. Needing to coordinate with non-associated medical offices to attain medical records or prescriptions can cause frustrations for the potential participant, medical office, and study team. In recruitment, onboarding, and trial maintenance, this study design relies on consistent communication to support participant enrollment and satisfaction.

**Trial registration:**

Treatment of Disturbed Sleep in Progressive Supranuclear Palsy (PSP); NCT04014389. Registered on June 2, 2019.

## Administrative information

Note: the numbers in curly brackets in this protocol refer to SPIRIT checklist item numbers. The order of the items has been modified to group similar items (see http://www.equator-network.org/reporting-guidelines/spirit-2013-statement-defining-standard-protocol-items-for-clinical-trials/).

**Table:** 

Title {1}	Treatment of Disturbed Sleep in Progressive Supranuclear Palsy: A randomized, remote, double-blinded, 6-week cross-over design study protocol comparing Zolpidem, Suvorexant and placebo.
Trial registration {2a and 2b}.	“Treatment of Disturbed Sleep in Progressive Supranuclear Palsy (PSP)”; NCT04014387; 6/19/2018
Protocol version {3}	Version 2 12/9/2019 WIRB^®^ Protocol #20181608 UCSF IRB: 17–23212
Funding {4}	Rainwater Charitable Foundation
Author details {5a}	Esther M Li, BS, Clinical Research Coordinator, Psychiatry UCSF Felicia Song, BS BA, Clinical Research Coordinator, Psychiatry UCSF Quentin Coppola, BS, Clinical Research Coordinator, Psychiatry UCSF; PhD Candidate, Northeastern University Leslie Yack, RPSGT, Sleep Technologist, Psychiatry UCSF; SFVA Michael M. Le, BA, Clinical Research Coordinator, Psychiatry, UCSF; DO Candidate, California Health Sciences University Samirah Javed, BS, Clinical Research Coordinator, Psychiatry UCSF; Program Manager, Google Deep Mind Program Natalie S Pandher, BS MS, Clinical Research Coordinator, Psychiatry UCSF Igor Prufer Q C Araujo, MD, Behavioral Neurology Clinical Fellow, UCSF Olga Mayzel, BS, Data Manager, Psychiatry UCSF; SFVA Hilary H Heuer, PhD, Professional Researcher, Neurology UCSF Mary Koestler, PhD, Nurse Manager, Neurology UCSF Bruce L Miller MD, Professor of Neurology, UCSF Adam L Boxer, MD PhD, Professor of Neurology, UCSF Lawren Vandevrede, MD PhD, Assistant Professor of Neurology, UCSF Lea T Grinberg, MD PhD, Professor of Neurology, UCSF Christine M Walsh, PhD, Associate Professor of Neurology, UCSF Thomas C Neylan, MD, Professor of Neurology & Psychiatry, UCSF; SFVA
Name and contact information for the trial sponsor {5b}	Thomas C. Neylan, MD Thomas.neylan@ucsf.edu University of California, San Francisco
Role of sponsor {5c}	Sponsor: Thomas Neylan, MD; Professor of Psychiatry, University of California, San Francisco. Dr. Neylan designed and is leading a UCSF team to administer all aspects of the study. Dr. Neylan will lead the public dissemination of the findings and report. Funder: Rainwater Charitable Foundation (RCF). The RCF funded this trial after a grant submission to them through the Tau Consortium. The RCF provided feedback on the grant submission with suggestions for how to improve the study design, however it was up to the sponsor on whether these suggestions should be implemented. The RCF is provided progress updates but does not have access to any identifiable participant data and is only provided de-identified summaries in progress updates.

## Introduction

### Background and rationale {6a}

Progressive supranuclear palsy (PSP) is a rapidly progressing atypical parkinsonism disorder characterized by the accumulation of primarily 4R tau tangles [1, 2] within the frontotemporal disease (FTD) spectrum, with a prevalence of 5.3 in 100,000 individuals [3]. PSP is phenotypically heterogeneous, presenting with motor and cognitive issues including gait and balance instability, bradykinesia, dysphagia, supranuclear gaze palsy, and executive dysfunction [4–7]. PSP phenotypic clinical subtypes include Richardson’s syndrome (PSP-RS) [8], primary progressive aphasia (PPA-PSP) [9], corticobasal syndrome (CBS-PSP) [10] and progressive akinesia with gait freezing (PAGF-PSP) [11]. Although treatment interventions for PSP to date have proved disappointing [12], increasing research on these various phenotypic profiles of PSP has led to more therapeutic clinical trials [13–15] primarily focused on PSP-RS. One therapeutic paradigm yet to be elucidated is the sleep-wake cycle in PSP [16].

PSP has distinct localizing features regarding disease onset, neuronal vulnerability, and progression [17]. Specifically, the hypothalamus and brainstem are among those regions earliest affected in the disease [18] and these general areas help regulate the sleep/wake patterns [19]. We found that individuals with PSP experience disrupted sleep with significant increases in wake after sleep onset and reductions in rapid eye movement (REM) and non-REM (NREM) sleep [20–22]. Following a night of little sleep, these individuals lack a propensity to sleep during the day and experience longer sleep latencies or hyperarousal in the multiple sleep latency test (MSLT) [22]. Typically, neurodegenerative disorders promote excessive sleepiness and daytime naps [23]. Daytime hyperarousal is rare in the absence of stimulant use or restless legs syndrome. The unusual combination of profound sleep deprivation without recuperation suggests that sleep homeostasis is disrupted in PSP.

Concordant with the diminished sleep observed in PSP, in neuropathological studies, we found that wake-promoting neurons (WPN) are relatively spared in PSP as compared to those with Alzheimer’s disease (AD) [24, 25]. A clinical feature of AD is increased sleep throughout the day, an opposite sleep/wake profile to PSP. In a clinicopathological comparison of nocturnal sleep/wake patterns and WPN across AD and PSP patients, PSP had more disrupted sleep and a greater number of WPN than AD, where neuronal count within WPN is associated with greater nocturnal wake, regardless of disease [17]. The WPN studied were the orexinergic neurons in the LHA, the histaminergic neurons in the TMN, and the noradrenergic neurons in the LC. The orexinergic neurons have previously been described as the sleep-on/off switch [26], and they are relatively spared in hyposomnolent PSP. Thus, an orexinergic antagonist may be more effective in treating sleep/wake issues than other hypnotic medications. Given the pattern of diminished sleep duration, hyperarousal, and the relative sparing of wake-promoting systems in PSP, we designed a protocol to test the safety and efficacy of a hypnotic medication that targets the orexin/hypocretin system (suvorexant) and contrast it to a standard benzodiazepine receptor agonist (zolpidem) which targets GABAergic sleep-promoting systems and placebo.

Suvorexant (Belsomra) is an FDA-approved, dual orexin receptor antagonist prescribed to treat insomnia. Phase 3 clinical trials examining suvorexant have found that, compared to placebo, suvorexant is effective for improving sleep onset and maintenance in elderly (15 or 30 mg/day) and non-elderly (20 or 40 mg/day) insomnia patients [27–29]. Zolpidem (Ambien) is an FDA-approved benzodiazepine receptor agonist that has been extensively studied for the treatment of insomnia in older adults [30]. Zolpidem has been examined in a small number of cases with PSP which demonstrated some transient effects on sleep and motor symptoms [31–35], but has not been studied in a controlled clinical trial. Suvorexant offers a novel approach targeting normal physiological mechanisms for sleep induction by selectively acting on wake-promoting orexin receptors to inhibit brain regions involved in wake regulation versus the generalized CNS suppression seen in Zolpidem.

Given PSP’s rarity, remote clinical trial methods can enhance recruitment by reducing travel burden and enabling participation from underserved areas nationally. This study aims to establish a remote trial protocol to improve accessibility, decrease costs, and facilitate broader engagement. A remote approach can accelerate recruitment and outcome reporting while encouraging participation from patients, providers, and industry stakeholders.

### Objectives {7}

1. To examine the efficacy and safety of suvorexant on objectively measured sleep and clinical global ratings of change in symptomatic severity in subjects with PSP.
  Hypotheses: (H_o_1a) Suvorexant is associated with increased sleep efficiency relative to placebo as measured with actigraphy and an ambulatory EEG monitoring device at baseline and during the 7 days of the study drug condition. (H_o_1b) Suvorexant therapy produces a greater change in clinical global impression of symptomatic severity relative to baseline at the end of the week of treatment compared to placebo.
2. To examine the efficacy and safety of zolpidem on objectively measured sleep and clinical global ratings of change in symptomatic severity in subjects with PSP.
  Hypotheses: (H_o_2a) Zolpidem is associated with increased sleep efficiency related to the placebo as measured with actigraphy at baseline and during the 7 days of the study drug condition. (H_o_2b) Zolpidem therapy produces a greater change in clinical global impression of symptomatic severity relative to baseline at the end of the week of treatment compared to placebo.
3. Compare and contrast the effects of suvorexant and zolpidem on efficacy and safety in subjects with PSP.
  Hypothesis: (H_o_3) Suvorexant, relative to zolpidem, produces greater improvement in sleep efficiency, greater changes in clinical global impression of symptomatic severity, and fewer adverse effects.
4. Test the acceptability and feasibility of conducting a remote randomized clinical trial using the Frontotemporal Dementia (FTD) disorders Registry. The FTD Registry is a comprehensive database that collects and tracks essential information about individuals with FTD disorders (including PSP), facilitating research and advancements in understanding and treating this neurodegenerative disorder.

### Trial design {8}

The design is a remote, double-blind, cross-over, within-subject 6-week trial, with 3 one-week-long conditions, each separated by 1-week washout periods (Table 1). The order of the 3 conditions is randomized and counterbalanced: placebo (microcrystalline cellulose), 15 mg/day suvorexant, 5 mg/day zolpidem.

**Table 1:**
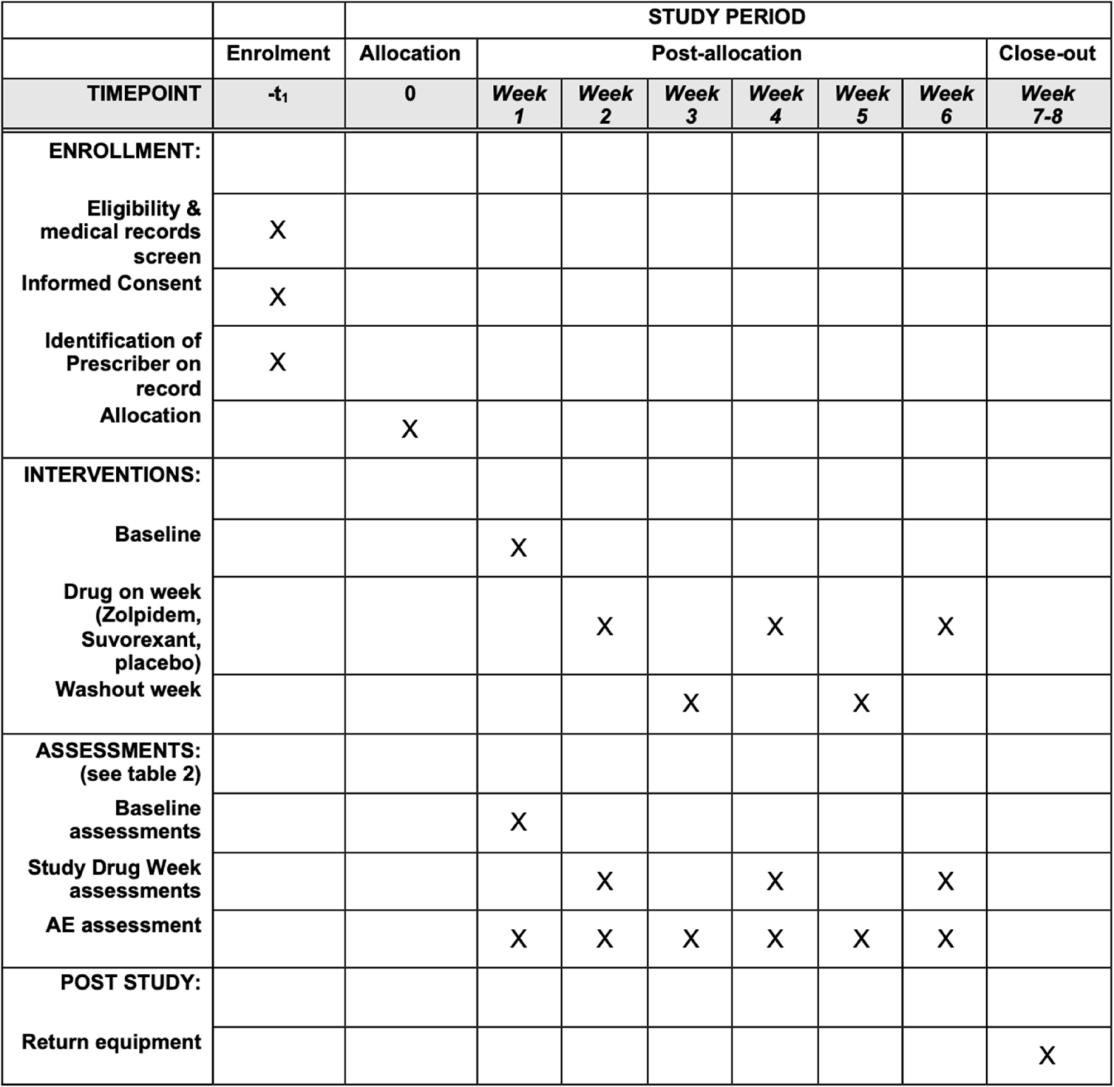
Schedule of enrolment, interventions, and assessments

**Fig. 1:**
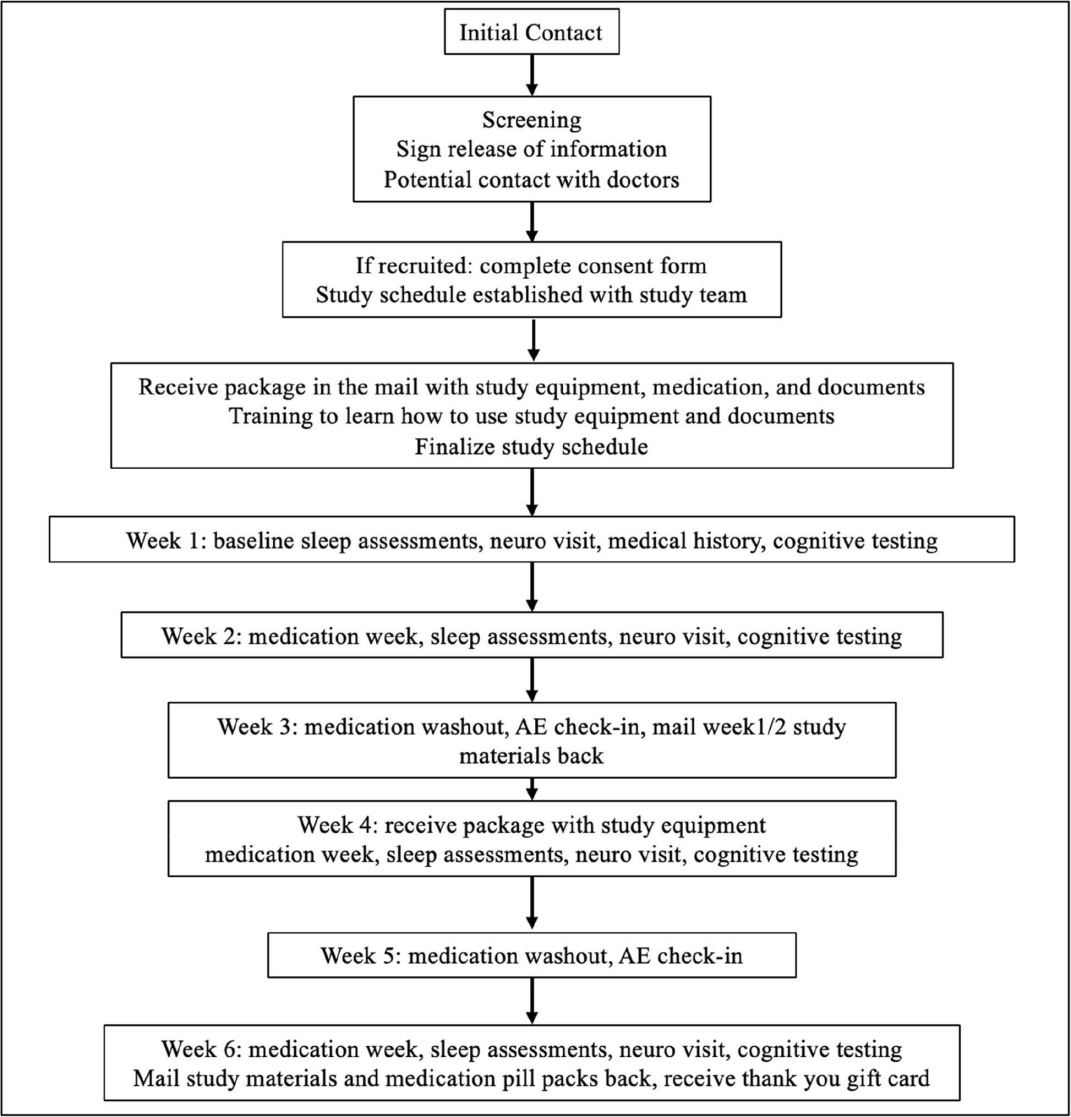
Flow diagram detailing steps of the onboarding process

* individual outcome details in Fig. 1 and Table 2

**Table 2:**
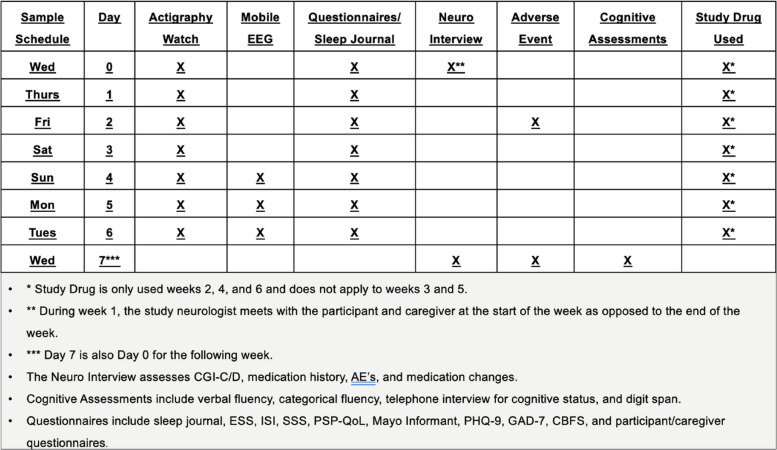
Detailed schedule during the baseline and three study drug weeks

## Methods: participants, interventions, and outcomes

### Study setting {9}

This is a fully remote clinical trial. Though the study is run through the University of California, San Francisco (UCSF), an academic hospital, participants do not attend any in-person assessments. The participant and caregiver are asked to complete all study measures and virtual meetings from their home. There are no other study sites. The UCSF study team obtains referrals from patient registries, support groups, community clinics, and academic hospital clinics within the USA. We also used a commercial IRB that created pragmatic advantages for conducting a national recruitment strategy.

### Eligibility criteria {10}

#### Inclusion criteria

1. Male or female ≥ 18 years of age at baseline.
2. Documentation of a progressive supranuclear palsy (PSP) diagnosis as evidenced by one or more clinical features consistent with the progressive supranuclear palsy clinical phenotype as described in the Movement Disorder Society criteria (Höglinger et al., 2017) or the NINDS-PSP criteria [6]. This diagnosis should be verified through co-enrollment in ALLFTD (Advancing Research and Treatment in Frontotemporal Lobar Degeneration (ARTFL) and Longitudinal Evaluation of Familial Frontotemporal Dementia Subjects (LEFFTDS)) Research Study. ALLFTD is an on-going, nationwide, longitudinal study for individuals and family members with frontotemporal disease spectrum, which includes PSP. As an alternative to co-enrollment in ALLFTD, the potential subject can show evidence of an accurate diagnosis of PSP to the satisfaction of the study team doctor (e.g., through review of medical records and/or specific communication with a known medical doctor).
3. Written informed consent (and assent when applicable) obtained from the subject or the subject’s legal representative and the ability for the subject to comply with the requirements of the study.
4. Have an active caregiver who is willing and able to participate in this study. A singular caregiver is consented as a study partner and stays consistent throughout the trial.
5. Have a mailing address.
6. Have access to a phone.
7. Have stable medications (aside from sleep-modifying medications) for 4 weeks prior to actively starting the study.
8. Be free of sleep-modifying medications for 1 week prior to actively starting the study.
9. Be willing to maintain a stable sleeping environment and their typical daily schedule for the duration of the 6-week study.
10. Resides in a US territory or state covered by our research study team.

#### Exclusion criteria

1. Are pregnant, breastfeeding, or unwilling to practice birth control if appropriate during participation in the study.
2. Presence of a condition or abnormality that in the opinion of the investigator would compromise the safety of the patient or the quality of the data.
3. Presence of a major psychiatric disorder aside from anxiety or depression.
4. Presence of a medical condition other than PSP that could account for cognitive deficits (e.g., active seizure disorder, stroke, vascular dementia).
5. Presence of current substance abuse or substance dependence.
6. Presence of a significant systemic medical illness (e.g., significant cardiovascular, hematologic, renal, or hepatic disease).
7. Presence of current medication likely to affect sleep outcomes: benzodiazepine receptor agonists (e.g., zolpidem), suvorexant, sedating antipsychotics (e.g., quetiapine), sedating antihistamines (e.g., Benadryl), low dose sedating antidepressants (e.g., trazodone, doxepin), over the counter sleep-inducing medications (e.g., Tylenol-PM), neuroleptics in the phenothiazine and haloperidol families which (1) the potential participant is not able/willing to stop taking for 1 week prior and for the 6-week duration of the study and/or (2) if removed could have a persistent effect beyond the 1-week washout period.
8. Presence of insulin-dependent diabetes.
9. History of mental retardation.
10. Unable to communicate with the research team.

### Who will take informed consent? {26a}

Trained clinical trial team members read through the consent forms with the participant and caregiver over teleconference Zoom, Inc. The study team member then answers any questions the participants may have about the study. Prior to signing the consent form, potential participants are asked a series of questions related to the study consent form which they read thoroughly and go through with the study team. If the participant is unable to answer the questions, their power of attorney/surrogate consent provider may provide consent unless the participant expresses resistance or dissent to participation, at which point they would be excluded from the study. The study care partner is also required to sign a consent form which they read thoroughly and go through with the study team. Both consent forms are required for enrollment.

### Additional consent provisions for collection and use of participant data and biological specimens {26b}

This trial does not involve collecting biological specimens. However, as part of the consent process, participants are informed that they may be asked to provide the UCSF study team with their medical records. Information gathered directly from the participant/caregiver by the researchers will be part of their research records but will not be added to their medical record. In the event of an adverse event (AE), information about their involvement in the study may be entered into their medical record. Participant information may be shared with doctor(s) that they have identified on signed release of information and HIPAA authorization forms, or with collaborators from other on-going studies that they are a part of. If the participant/caregiver indicates that they wish to be contacted for additional studies (related to sleep/wake patterns, clinical trials, or research in general), their information may be shared for those studies to contact them. The participant/caregiver’s personal information may be given out if required by law. If information from the study is published or presented at scientific meetings, only de-identified information would be used. Authorized representatives from the following organizations may review participant research data for the purpose of monitoring or managing the conduct of the study:

- Representatives of the funding agency (Rainwater Foundation)
- Representatives of University of California
- Representatives of the Food and Drug Administration (FDA)
- Representatives of studies you are/were enrolled in (e.g., ARTFL/LEFFTDS/ALLFTD/4RTNI)
- Representatives of the Western Institutional Review Board (WIRB)

Potential participants go through the screening questions on the phone and/or through the study-specific website which has a link to Qualtrics with the screening questions to provide confidential responses to the research team. Prior to starting the screening questions, participants will either give an oral consent over the phone, or an electronic indication of consent through Qualtrics if accessing the screening questions through the study-specific website. The screening questions cover the inclusion/exclusion criteria to determine if the potential participant passes the criteria and is potentially eligible for the study. As part of the screening process, potential participants are asked to indicate which of the following studies: ARTFL/LEFFTDS/4RTNI/ALLFTD they are enrolled in and which site they attend. This information will be gathered so that the participant’s diagnosis of progressive supranuclear palsy can be confirmed. Participants may be asked to complete a release of health information form. If the participant is not a participant in ARTFL/LEFFTDS/ALLFTD/4RTNI, the potential participant will be asked to provide their medical record for the study doctor to review. After the diagnosis can be confirmed to the satisfaction of the study research team, the potential participant is consented.

## Interventions

### Explanation for the choice of comparators {6b}

The primary objective is to assess the clinical efficacy as measured by the change in sleep and general functioning of an individual following 7 days of treatment with two FDA-approved hypnotic drugs as compared to placebo. The secondary objectives are to determine whether suvorexant compared to zolpidem is more effective for sleep disturbances in PSP and to test the acceptability of and feasibility of using the web and telephone-based methods for recruiting, consenting, and interacting with participants with PSP.

As previously described, PSP is a relatively rare disease with considerable hyposomnolence. The selected hypnotics are expected to show effects within 7 nights. To reduce the number of participants and leverage the fast-acting FDA-approved medications, a cross-over study design was chosen. The study medications selected were 15 mg/day suvorexant, 5 mg/day zolpidem, and placebo (microcrystalline cellulose).

### Intervention description {11a}

#### Study drug preparation

The research pharmacist prepares identical appearing suvorexant (15 mg/day), zolpidem (5 mg/day), and placebo (microcrystalline cellulose) capsules in a smart blister pack containing 3 rows, 1 for each week of study medication. The order of receiving suvorexant, zolpidem, or placebo (6 different possible order sequences) is randomized in blocks of 6 to ensure equal distribution of the 6 possible sequences. Only the research pharmacist knows the randomized order, and not the main study team. The unblinding of the study groups will not take place until after a full database lock when all clinical ratings and measures of sleep have been completed and databased. For more information on dosage and preparation, see section {16c}.

### Participant involvement

The initial contact and onboarding process before the participant starts the study is diagrammed in Fig. 1. A schedule of events representing the required testing procedures performed for the duration of the study is diagrammed in Table 2. Prior to conducting any study-related activities, written informed consent and the Health Insurance Portability and Accountability Act (HIPAA) authorization must be signed and dated by the participant or participant’s legal representative and caregiver. If appropriate, assent must also be obtained prior to conducting any study-related activities. Patients who have consented to the protocol are mailed a package that contains (1) a printed booklet including a study schedule, FDA-approved medication information, a sleep journal, and symptom rating scales; (2) an actigraphy watch that tracks light and circadian rhythms (Philips Spectrum actigraph, Philips Inc., Cambridge, MA); (3) a mobile electroencephalography (EEG) device (Sleep Profiler, Advanced Brain Monitoring Inc., Carlsbad, USA); and (4) a smart blister pack that records the time of pill removal (Med-ic, Ontario, CA) with 3 separate, 1-week rows of study medication.

The package sent contains return mailers that enable the patient/caregiver to return the mobile EEG, movement sensor, printed booklet, and the blister pack with any unused medication. Two packages are sent to the patient during the study, with the second arriving during week 3 with an additional mobile EEG and return mailer. Additional packages may be mailed to and from the participant/caregiver as needed. The clinical research coordinator makes a schedule of virtual visits (i.e., Zoom or telephone). Participants are given reminders of when to start the study medication at the end of each washout week. All study drugs are to be taken at bedtime. Assessments conducted during the baseline period are the reference for the 3 study conditions (suvorexant, zolpidem, and placebo). The protocol for both the washout weeks, baseline condition on no hypnotic medications, and study drug weeks is shown in Table 2.

Drug administration: each of the 3 identically appearing compounded study drugs (suvorexant, zolpidem, and placebo) is supplied in a smart blister pack which produces a timestamp when a pill is removed. The study drug is taken at night for 7 nights in a row. The following 7 nights is then a study drug-free week, followed by the second study drug for 7 nights. Subsequently, there is the second and final study drug-free week, followed by the third and final study drug for 7 nights.

Treatment compliance: participants and their caregivers are instructed to take/administer the oral study drugs at bedtime each night during the study drug weeks. The blister pack generates a timestamp when the medication is removed. Participants are instructed that there should be no delay between removing the study drug from the blister pack and the time of actual administration. However, as part of the sleep diary, the time of actual administration and the duration of the delay from drug removal from the blister pack and administration is queried each night. At the end of the study, the blister pack is returned by mail to the UCSF study team. Adverse events are noted on the sleep diary and queried over the phone/Zoom by the study team.

### Criteria for discontinuing or modifying allocated interventions {11b}

The 6-week study includes two active drug weeks (one for each FDA-approved drug). During drug-on weeks (2, 4, and 6), the study team contacts participants/caregivers twice weekly to monitor AEs, and once weekly during non-drug weeks (3 and 5). In the event of an AE, the neurologist assesses the severity and degree of relation to the study medication. If an AE is determined to be severe and due to the medication, the participant would cease drug administration and be assessed for fitness to continue the other conditions or halt participation in the trial entirely. The medical monitor and the data and safety monitoring committee (DSM) are available to oversee the study.

Prior to starting the study, the patient/caregiver is in contact with the study team to learn how to use the equipment and is instructed about using the study packet. These instructions include techniques to reduce discomfort while wearing any of the devices and how to minimize sleep disruption while wearing them.

If skin irritation occurs from the sensors when using the mobile EEG, the participant/caregiver would be asked to contact the study team to discuss whether the participant would prefer to discontinue measurements with this device. If at any time the participants/caregivers indicate that they are uncomfortable with the questions posed or with any part of the study, the study team discussed the concern to determine if they wish to continue and whether their discomfort can be mitigated. If participants/caregivers cannot spend long periods on the phone/Zoom at any one time, assessments can be done individually on the days of testing to provide breaks.

All personnel are up to date on their human research Collaborative Institutional Training Initiative (CITI) training and have experience working with participants. Prior to starting the study, study team members discuss with the patient/caregiver a plan for where the participant would go in case of a serious AE (SAE). In the case of an AE, the medical monitor would work with the study team to determine the best course of action. Prior to starting the study, participants complete a personal health information (PHI) release form so that the UCSF study team can communicate with their identified local doctor if an AE were to occur that requires local treatment/oversight.

### Strategies to improve adherence to interventions {11c}

Prior to starting the study, the participant/caregiver is sent a timetable with dates for when the key study events occur (e.g., on nights 4, 5, and 6 the participant wears the mobile EEG device; on day 7 the participant meets with the neurologist). In addition, the participant/caregiver is given reminders on the day of key events (e.g., “please start your study medication tonight”). The clinical trial coordinator and study trial doctor have frequent contact with the participant and caregiver throughout the 6-week trial. Specifically, the participant/caregiver has a check-in, reminder, or assessment at least once every 2–3 days over the course of the 6-week study. Further, the use of the medication blister pack, actigraph, and mobile sleep EEG device has timestamps through which the study team can determine if they were used at the correct times throughout the study. The participant/caregiver returns study equipment through the mail twice throughout the study: (1) at the end of the second medication week (week 2) and (2) at the end of the study (week 6). Once the second box of study equipment is received, the study team sends a study payment of $125 to the participant/caregiver. Overall, the practices utilized and described here help support adherence to the study protocol.

### Relevant concomitant care permitted or prohibited during the trial {11d}

Participants are prohibited from taking any sleep-altering medication 1 week before the trial and any non-study-related hypnotics during the trial. All other concomitant care is allowed throughout the study.

### Provisions for post-trial care {30}

#### Transition from research participation

Participants will return to their usual standard care immediately following the protocol. Should the participant experience harm from their participation, UCSF would provide necessary medical treatment.

### Outcomes {12}

A within-subject 3-treatment cross-over design is being utilized for this study. The 6 possible sequences of study medications are randomized in blocks of six to reduce the likelihood of order/carryover effects. The primary outcome measures for aims 1–3 are baseline-post treatment change in sleep efficiency (as measured by actigraphy), the Clinical Global Impression Change score (CGI-C) [36, 37] which produces a single change score referenced to the baseline Clinical Global Impression Disease Severity score (CGI-ds) [36, 37], and the number of adverse events. Thus far, no actigraph, including the Philips Actigraph, has been validated in this population. The use of actigraphy not validated in PSP is a limitation of this study which we attempted to minimize through the addition of EEG. For objective 4, the outcome measures are the proportion of individuals who found the study through the FTD Registry vs. other identified sources that (1) completed the study screener and (2) consented to participate in the study. Secondary outcome measures for objectives 1–3 are wake after sleep onset (WASO, Sleep Profiler device), total sleep duration (Sleep Profiler), subjective reports of sleep duration and sleep quality acquired through the sleep journal, as well as changes in insomnia severity (Insomnia Severity Index (ISI)), participant/caregiver rating of beneficial vs. adverse events, sleepiness (Stanford Sleepiness Scale (SSS) and Epworth Sleepiness Scale (ESS)), and alertness (question 8 of the Mayo Informant questionnaire). Other outcomes are Cortical Basal Functional Rating Scale (CBFS), PSP quality of life (PSP-QoL), executive function (composite of digits backward, categorical fluency, and letter fluency), mood on Patient Health Questionnaire-9 (PHQ-9) and General Anxiety Disorder-7 (GAD-7), concomitant medication use, and medical history. Each outcome measure for objectives 1–3 will be assessed as change from baseline (week 1). See Table 2 for timing of the outcomes assessed.

### Participant timeline {13}

Potential participants complete the online eligibility survey. Once completed, they are contacted by a member of the study team within 24 h to begin enrollment. When medical records are received and a diagnosis of PSP is confirmed by the study neurologist, the coordinator schedules a meeting by phone or Zoom to complete an informed consent with the participant and caregiver. Subsequently, as controlled medications cannot be prescribed by a physician licensed in a state other than the state a patient is residing in, a pharmacy order form is completed either by the study principal investigator if the participant resides within the study state (CA, USA) or the participant’s physician if the participant resides out of the study state (outside of CA but within the USA). Medications are ordered and filled from the pharmacy once PHI release, medical records, HIPAA authorization for research, consent, and pharmacy order forms are obtained.

Once all onboarding forms are complete, participants and caregivers are in contact with the study coordinator to establish a study schedule for the 6 weeks in trial. The participant and caregiver are in contact with the study coordinator to be trained on how to use the equipment and how to follow the protocol paperwork. Neurologist visits and cognitive tests are scheduled and performed according to the process described in the “Assessments” section above. At the end of week 2, the participant mails out the first package with the respective devices and paperwork while keeping the medication pill packs until the end of the study. A similar box containing week 4 and week 6’s study materials is mailed by the study coordinator during week 3. Each active study week, the participant and caregiver are doing over the phone/Zoom assessments for no more than 90 min (cognitive testing, assessments, and interview with the study neurologist; and an extra 30 min on baseline week for medical history intake). The questionnaire packet takes approximately 60 min to complete per week. The mobile EEG device is worn for 3 nights of each active study week, and the actigraph is worn for ~24 h/day for 7 days a week. At the completion of week 6, the participant/caregiver returns all the study materials via mail to the research team. Once this final package is received, the study coordinator sends the patient their study payment of $125.

### Sample size {14}

To test comparisons of each active drug vs. placebo, we expect a sample size of *N* = 60 primary participants to yield a power of 0.80 with alpha = 0.05 to detect a standardized effect size of *d* = 0.32. A caregiver is required for each primary participant in this study to do assessments and to help the primary participant to complete the required tasks. While the overall sample size is modest, the likelihood of detecting meaningful differences is supported by prior publications in PSP populations, where small sample sizes have still yielded significant differences (e.g., [38]) [27]. Therefore, the goal of this study is to recruit a total of 120 participants: 60 primary participants and their caregivers. Our actual enrollment was 80 participants: 40 participants and their caregivers.

### Recruitment {15}

Members of the study team search for potential participants through patient records of those seen by the investigators or patients seen within the UCSF Memory and Aging Center. We also leverage multiple avenues of recruitment from outside organizations that advertise our study to its members (i.e., CurePSP, FTD Registry) through online posts, highlighting the study in their newsletters or through targeted emails. Online posts can include posts on their website, Facebook, and Twitter accounts briefly describing the summary and providing a link to the study website.

The study team also sends letters to providers who specialize in PSP to share our study information with potential candidates. Participants with a diagnosis of PSP participating in related studies are also referred if they have previously indicated interest in learning about other studies. Similarly, the study is also presented to other investigators and within the community by the study research team and affiliates.

After the potential participants have either contacted us (through email/phone/in person) or been identified, they may be emailed or called by a study team member or directed to our study website. Communications utilized text submitted and approved by the IRB, describing the study to potential participants.

The caregivers either self-identify or are identified by the primary participant during the primary participant’s initial contact or screening process.

## Assignment of interventions: allocation

### Sequence generation {16a}

The order of receiving suvorexant, zolpidem, or placebo (6 different possible order sequences) is randomized in blocks of 6 by the UCSF research pharmacist to ensure equal distribution of the 6 possible sequences. The randomized blocks will remain with the UCSF Investigational Drug Service for the duration of the study; thus, randomization is conducted without any influence from the principal investigators, coordinators, or participants.

### Concealment mechanism {16b}

The order of the medications follows a randomization key that is done by the research pharmacist; allocation concealment is ensured throughout the study, as the unblinding of the study groups will not take place until after a full database lock of all clinical ratings and measures of sleep has been completed and databased. Study medications and matching placebo are encapsulated in appropriate, standard-sized 3 gelatine capsules, with sufficient opacity and color to hide the contents and not disclose any possible difference between the 2 medications and placebo.

### Implementation {16c}

The research coordinator (RC) is the initial line of contact with participants and their caregivers throughout recruitment and study training. The RC also schedules all virtual meetings during the study and creates email reminders for both the participant and caregiver, as well as the study neurologist.

Valor Compounding Pharmacy (formerly Wellspring Compounding Pharmacy) in Berkeley, CA, provides identical appearing suvorexant (15 mg), zolpidem (5 mg), and placebo (microcrystalline cellulose) capsules. The UCSF research pharmacist directly receives all study drugs from Valor Pharmacy and stores them in a limited-access area under appropriate environmental conditions. The research pharmacist develops the randomization order and has the unblinding key.

Once the participant has been consented, the study coordinator assigns them a participant ID number. The study coordinator then provides the pharmacist with the participant ID; the pharmacist prepares a smart blister pack that contains three columns, a column for each week of study medications, labeled for each week they will be taken (week 2, week 4, week 6). Once a member of the study team picks this up from the UCSF pharmacy, it is packaged with the necessary study equipment and paperwork for the entire study and mailed to the participant and caregiver.

## Assignment of interventions: blinding

### Who will be blinded {17a}

The pharmacist is the only unblinded individual and has no contact with the participants. All other study personnel, the participant, care partner, and medical providers are blind to the condition in any of the study drug-on weeks (weeks 2, 4, 6).

### Procedure for unblinding if needed {17b}

Only at the end or termination of the study will the study team be unblinded. Due to the cross-over design and the use of FDA-approved medications in the trial, in the case of an SAE, study personnel can remain blinded and provide the treating physician with the names and doses of the two potential therapeutic medications.

## Data collection and management

### Plans for assessment and collection of outcomes {18a}

All questionnaires and the sleep diary are administered using a printed study booklet that is filled out by both the patient and caregiver. Sleep is assessed using actigraphy and a mobile sleep EEG device. Medication administration timing is captured using a smart blister pack. All other study metrics are assessed through interview or assessment over the phone or Zoom. Assessments conducted at the end of the baseline period (week 1) are the reference for the 3 study conditions. The timing of the assessments for both baseline and study drug weeks (weeks 2, 4, and 6) is shown in Table 2.

### Training of assessors

New RCs onboarded to manage the trial shadow a current RC through the specific set of protocols from trial start to end. In the early phases of training, cognitive tests, data entry, and other encounters are co-scored and monitored to ensure data quality.

Adverse events are assessed on day 2 and day 7 of baseline week and weeks 2, 4, 6. During weeks 3 and 5, AEs are assessed according to participant availability, usually on day 2 or 3.

Medication changes are obtained at the same time points as the four CGI assessments by the study neurologist.

Medical history is obtained during baseline week.

Clinical Global Impression of Disease severity (CGI-ds) (baseline evaluation) via Zoom or telephone: On day 7 of the baseline week, a study neurologist completes a structured interview with both the patient and caregiver present that covers relevant medical/neurological history, observation/appearance, mental/cognitive state, behavior, sleep/appetite, psychomotor activity, social behaviors, daily activities, basic functions, speech and swallowing, visual problems, rigidity/stiffness, and gait. These symptoms are rated on a 1–7 scale (normal to extremely impaired) and used for the overall impression of disease severity, a 1–7 scale (normal to extremely ill) [36, 37].

Clinical Global Impression Change (CGI-C) via Zoom or telephone: On day 7 at the end of the 3 active treatment conditions (weeks 2, 4, and 6), a study neurologist does a follow-up assessment similar to the CGI-ds and produces a final rating of overall impression of change as rated on a 1–7 scale centered on 4 which designates no change. A score of 1–3 describes marked, moderate, and minimal improvement. A score of 5–7 describes a minimal, moderate, and marked worsening [36, 37].

Movement sensors: Participants wear a wrist actigraphy (Spectrum Plus, Phillips Respironics Inc.). Once all data collection is completed, data are analyzed for sleep onset/offset times, estimated total sleep time, sleep efficiency, and wake after sleep onset. This sensor is worn by participants during the baseline week and during each of the 3 study drug-on weeks.

Mobile EEG sleep assessment: Patients are asked to wear a simple mobile EEG device (Sleep Profiler, Advanced Brain Monitoring Inc.) which the caregiver may need to help place on their forehead for the last 3 nights of the baseline week and on the last 3 nights of each of the 3 study drug-on weeks.

Sleep journal: Participants (assisted by the caregiver as necessary) complete a daily report on subjective sleep, daily habits, sleep/wake times, questions related to protocol adherence, and how the participant feels. This is completed during each day of the baseline week and during each day of the 3 study drug-on weeks.

Cognitive assessment: Assessments are done by phone or over Zoom by the study coordinator, where participants complete versions of the Telephone Interview for Cognitive Status, categorical fluency, letter fluency, digits forward, and digits backward on the last day of the baseline week and each of the 3 study drug-on weeks.

Questionnaires: On the last day of the baseline week and each of the 3 study drug-on weeks, participants and caregivers complete a series of questionnaires. Within the questionnaire booklet is as follows: (1) mood assessments: participants complete the PHQ-9 [39] and GAD-7 [40] to assess depression and anxiety; (2) satisfaction assessment: participants and caregivers are given the Treatment Satisfaction Scale; (3) alertness: caregivers are asked to complete an assessment of the participants’ daytime alertness using question 8 from the Mayo Informant—Sleep Questionnaire [41]; (4) sleep measures: participants are asked to complete the Epworth Sleepiness Scale [42] and the Insomnia Severity Index (ISI) [43]; (5) “daily living assessment”: participants are given the CBFS [44] assessing behavioral, language, and cognitive impairments; (6) PSP quality of life [45] is also given to assess the quality of life of people with PSP. Participants are also asked to complete the Stanford Sleepiness Scale 3 times (9 am, 1 pm, and 5 pm) [46] the last day of the baseline week and each of the 3 study drug-on weeks. In addition, the study partner is asked to complete the Mayo Informant—Sleep Questionnaire in its entirety during the baseline week. On the final day of the study (day 7, week 6), the participant and study partner are also asked to complete a study satisfaction scale, which is a customized, study-specific questionnaire using a 7-point Likert scale.

### Plans to promote participant retention and complete follow-up {18b}

The length of the onboarding process depends in part on the speed at which necessary document transmission occurs between researchers and the potential participant’s neurologist or primary care physician. During onboarding, researchers rely on weekly scheduled contact with potential participants to maintain interest in the study.

All participants who complete the trial are offered a follow-up virtual meeting facilitated by either the PI or the study doctor, with the participant and caregiver or the participant, caregiver, and the participant’s doctor. Although the exact sequence of medications is undisclosed, information about medication dosing and sleep hygiene may be offered.

Once the trial has been completed, the research team will disseminate any research findings published in a peer-reviewed journal to all the participants who took part in the study.

Participants also receive payments of $125 total for completion of the 6-week trial. This is less than $21/week, a relatively modest amount of compensation that should not be coercive to any financially disadvantaged participants.

### Data management {19}

All identifying materials are kept in a secure, password-protected, limited-access database. Paper documents are stored in a locked location, separate from other data collected. Information is not shared without the participant’s prior approval. While PHI is used in the study, no PHI is used or transferred to any outside organization. All participant-related activities are done through UCSF. Participants are asked during the informed consent if they would like to be referred to other possible research studies in our research groups, and their contact information may be shared for referral purposes.

Mobile EEG devices are initialized at UCSF, and data from the mobile EEG are stored on the device company’s database once received back by the RC via mail (FedEx). Participants do not need to transfer data themselves. For example, the Sleep Profiler stores EEG, EOG, and EMG signals on the Sleep Profiler Advanced Brain Monitoring database so that we can use the proprietary algorithms to capture and process these signals to identify sleep. All data are de-identified, with minimal additional data shared. The data shared include year of birth, gender, and study condition. All questionnaires, CGIs, cognitive tests, and actigraphy data are stored on the UCSF secured, password-protected servers once received. Data entry is completed and validated by the RCs.

### Confidentiality {27}

Information gathered directly from the participant by the researchers is part of the research records and are not added to the participant’s medical record. If an adverse event occurs, information about the participant’s involvement in the trial may be added to the participant’s medical record. Medical records collected during the onboarding process are only shared within the research team. Data collected through this study may be shared but will first be de-identified. All publications will only present on de-identified data. As described earlier in the section: “Additional consent provisions for collection and use of participant data and biological specimens,” identifying information may be shared by law or by the participant’s behest. Further, listed, specific organizations that may look at and/or copy medical records and research data for research, quality assurance, data analysis, and monitoring or managing the conduct of this study.

### Plans for collection, laboratory evaluation, and storage of biological specimens for genetic or molecular analysis in this trial/future use {33}

N/A.

## Statistical methods

### Statistical methods for primary and secondary outcomes {20a}

The principal data analytic technique will be intent-to-treat analyses using random intercepts linear mixed models for repeated measures. Predictors in the models will include treatment, period (first, second, or third week on study drug), and treatment × period interaction (to test for unexpected order and carryover effects). Baseline scores on outcome measures will be included as covariates. Additional covariates will include disease severity (CGI-ds) and age. Potential missing data can be accommodated by the mixed model under the assumption of missing at random (MAR).

Safety analysis: The safety analyses population includes all randomized subjects who have taken at least one dose of study medication. These analyses will include those subjects who have reported an adverse event as well as those who do not report an adverse event. The incidence of treatment-emergent adverse events, type of adverse events, and frequency of withdrawal due to an adverse event will be summarized by treatment group. Appropriate statistical comparisons will be made between treatment groups in terms of (1) rates of adverse events, (2) discontinuation due to adverse events, and (3) adverse effect scales. Frequencies will be analyzed using the chi-square or Fisher exact test. The scales will be examined using nonparametric analyses when appropriate.

For objective 4, use of the FTD Registry will be assessed as successful if: participants report a high satisfaction rate with using the registry (>80% with a score of 4 or higher on a 5-point Likert scale captured on the study satisfaction questionnaire given on the last day of the study), and would recommend participation in registry-based research to patients and families with PSP (>80%).

### Interim analyses {21b}

Given the short duration, within-study design, and utilization of FDA-approved medications, we do not plan to do an interim analysis.

### Methods for additional analyses (e.g., subgroup analyses) {20b}

Overall, PSP-RS is easier to diagnose than the other PSP subtypes. Given the rarity of the disease, the relatively low sample size, and based on the likely disproportionate enrollment of PSP-RS vs. other PSP subtypes, we will not perform subgroup analyses.

### Methods in analysis to handle protocol non-adherence and any statistical methods to handle missing data {20c}

As mentioned earlier, any missing data can be accommodated by the mixed model under the assumption of missing at random (MAR).

### Plans to give access to the full protocol, participant-level data, and statistical code {31c}

All de-identified data collected during this study will be available for review upon formal request as in line with UCSF MAC Open Science data sharing policy. All data will undergo regulated institutional procedures including a formal transfer agreement to which the requesting party will need to provide their name, affiliation, and brief description of their analysis plan (The UCSF MAC Open Science Resource Request Application can be found here: https://ucsf.co1.qualtrics.com/jfe/form/SV_01EHhRABhqgPgO2). The protocol in its entirety will be made fully available upon direct request to the PI (TCN). The study team (particularly CMW, LV, ALB, TCN) would also be available to provide helpful information to other investigators that are attempting a similar remote clinical trial to assess the feasibility and efficacy of an FDA-approved medication to specific groups.

## Oversight and monitoring

### Composition of the coordinating center and trial steering committee {5d}

Principal investigator (PI): the PI meets with the study manager and study research coordinator for updates 1/week and is available for contact about an active or potential participant as needed. The PI signs the pharmacy request forms for participants residing in-state (within California). The PI and study manager oversee the budget and prepare bi-annual reports to the funding agency (Rainwater Charitable Foundation). The PI and study manager are responsible for study communications. Protocol deviations are approved by the PI, study manager, or medical monitor.

Study manager: the study manager meets with the PI and the study research coordinator for updates 1/week and is available for contact about an active or potential participant as needed. The study manager supports participant recruitment by (1) giving presentations to inform about the study and (2) working with community patient groups, organizations, and other PSP-focused research or medical groups throughout the USA. The study manager oversees the study protocol and general study management.

Research coordinator: a research coordinator is responsible for the enrollment of participants including contacting participants’ doctors to obtain medical records, performing the informed consent, and developing a trial schedule. The research coordinator schedules all telehealth check-ins throughout the trial as well as manages the shipping of study materials to the participant. The research coordinator does the cognitive assessments, manages the data, and is responsible for communicating to the PI, study manager, or medical monitor any potential protocol deviation, and documenting protocol deviations when they do occur.

Study neurologist: the study neurologist reviews medical records, confirms participant eligibility, and meets with the participants and caregivers throughout the study. The study neurologist works closely with the study research coordinator.

Back-up research coordinator: this research coordinator supports the primary research coordinator as needed.

Back-up study neurologist: this neurologist supports the primary study neurologist with participant assessments as needed.

Data and safety monitoring committee (DSMB): the DSMB is comprised members of the MAC, both directly and indirectly related to this trial. The DSMB will be updated on the study every 6–12 months.

Medical monitor: the medical monitor is responsible for the interpretation of any AE that may occur throughout the study, determining the severity and relation to the trial medication. They communicate with the study research coordinator, study manager, and PI as needed.

Study pharmacist: the study pharmacist did the initial randomization and holds the randomization key. They receive the study drugs and prepare the smart blister packets for the participants.

Study sleep technologist: the sleep technologist trains the research coordinators on scoring data from the mobile sleep EEG device and oversees the associated data quality.

### Composition of the data monitoring committee, its role and reporting structure {21a}

The trial maintains a medical monitor in case of adverse events. Participants have direct access to the PI 24 h/day in case of an immediate AE that does not warrant contacting the emergency services. All AEs and any deviations in study protocol will be noted and reported to the Data and Safety Monitoring Board (DSMB). The DSMB is comprised clinical researchers related and unrelated to the trial. The study team meets with the DSMB every 6–12 months to report any AEs and study deviations as well as recruitment updates.

### Adverse event reporting and harms {22}

The study team is in contact with the participants and caregivers twice a week to monitor for AEs. If at any time the study neurologist is concerned about the participant continuing, they will be removed from the study. The medical monitor and the DSMB are available to oversee the study.

In the case of an AE, the medical monitor will work with the study team to determine the best course of action. Prior to starting the study, participants complete a PHI release form so that we can communicate with their identified local doctor if an AE occurs that requires local treatment/oversight. Any deviations from the study protocol are assessed by the study team and documented.

### Frequency and plans for auditing trial conduct {23}

No independent audit of trial conduct is planned.

### Plans for communicating important protocol amendments to relevant parties (e.g., trial participants, ethical committees) {25}

Any changes in the protocol are submitted to the external IRB (Western Institutional Review Board, Inc.; Puyallup, WA, USA). Once approved, all study personnel, both the research team and any active participants, are updated as needed.

### Dissemination plans {31a}

Prior to starting the trial, participants are provided with copies of a brief study summary sheet with the names and doses of the medications provided in the study, along with the contact information of the study researchers. This summary sheet may be relayed to the participant’s doctor or presented to a medical team in case of an AE.

Upon trial completion, results will be published in a peer-reviewed journal and disseminated to all participants who were a part of the trial.

## Discussion

Administering a fully remote clinical trial faces unique issues that would otherwise be absent from in-person studies.

Recruitment: with a relatively rare disease, recruitment can be challenging. Though this study’s design is made to maximize the reach and accessibility of this study, recruiting for rare diseases, particularly for a symptom rather than disease-modifying trial, can be a challenge when other potential therapeutics may be in the pipeline. Organizations like ALLFTD and CurePSP are supportive and aid in recruitment. Collaborating with external sites or clinicians from the outset would have been helpful to potentially overcome delays in sending medical records to an out-of-state institution.

Further, recruitment is hampered by trying to identify a prescribing doctor for the study medications for outside of CA residents. Establishing other sites could help overcome or minimize the challenge of having an in-state doctor prescribe the study medications; however, this would involve those doctors prescribing study medications for participants they may not have evaluated in person and the subsequent medical-safety insurance-related uncertainties.

Onboarding: the onboarding process involves 2 key interactions with the potential participant’s doctor, obtaining the potential participant’s medical records and having the doctor sign a pharmacy order form that is sent to the UCSF Investigational Drug Pharmacy. In the case of an internal referral from UCSF, necessary paperwork is easier to complete, with medical records easier to access. Moreover, the pharmacy order form can be signed by the study PI, as approved by our IRB. In the case of out-of-state participants (individuals not residing within CA, USA), members of the study team must contact the potential participant’s doctor’s office and send all documents via secure fax. Often, this process takes multiple months to complete due to several factors including busy offices missing the fax or taking a long time to send the medical records in, or apprehension and uncertainty expressed by the potential participant’s doctor. The research coordinator may spend considerable time trying to gain access to these documents; potential participants can become frustrated with the delay, particularly as reviewing the medical records is a part of the eligibility screening process. Once a participant is eligible, there may be a further delay if the participant’s doctor needs to be the prescriber on record for the study drugs. Getting the scripts signed and sent to the pharmacist can again be a lengthy process, at times requiring alternate doctors to be the prescriber on record who is identified by either the participant or a study team member. This process typically takes around 3 months to have the participant fully enrolled thus far. It is crucial that the research coordinator maintains contact with the potential participant during the entire onboarding process to help reduce the potential participant’s frustration and to stay abreast of any changes that may be occurring for the potential participant and their potential study partner. Early partnerships, such as with PSP Centers of Excellence and prescribers in high-population states, would have helped streamline the onboarding process.

Remote data collection: remote data collection, as stated, is facilitated via rapidly delivered postage through FedEx. Although participants did confirm with a member of the research team or a researcher at the MAC, often times it was of paramount importance to confirm addresses over the phone. Missing or delayed packages can present a unique challenge given the sensitive nature of the pharmaceuticals. With the remote nature of this study, it is necessary to have additional check-ins and reminders to ensure participant safety, engagement, and data collection (ask the participant to check their actigraph wrist-band face to ensure the actigraph is still on, confirm that a participant and study partner have completed their questionnaire packet) and to prevent forgotten protocol steps (e.g., using the mobile sleep EEG device on particular nights). It can help to schedule package pick-ups to prevent participants/study partners from not returning the packages in a timely manner. Further, with a remote data collection approach, until the packages are received, it is not possible to know if the mobile sleep EEG device has successful recordings on it.

Data maintenance: data preservation presents a unique issue not only because this is a fully remote study, but because of the specific devices themselves. Members of the study team extensively explain and provide instructions for the devices that participants wear (e.g., written instructions, access to a pre-recorded standardized video, and training with a research coordinator over Zoom). However, despite the research team’s multiple weekly check-ins, which include verifying device functionality, these devices remain susceptible to malfunctions or incorrect use, potentially resulting in lapses in data collection. For example, the Sleep Profiler is a device that requires strict charging and set-up each night. Adhesive electrodes are to be snapped into the device and replaced each use as well as at least 3 h of charging time needed before data collection. Any deviations in the set-up protocol for the device can lead to bad or missing data out of the control of researchers. The watch holds an impressive charge that lasts multiple weeks at a time; however, if the device runs out of battery before either a participant or researcher notices, the result is missing data that may affect key measures of sleep. Due to these potential device issues, the importance of increased contact between members of the study team and participants is greatly noted.

## Trial status

Treatment of Disturbed Sleep in Progressive Supranuclear Palsy (PSP); NCT04014387; 6–19–2018.

Protocol version 2; 12-9-2019.

Start: 2019-06-02.

Study completion date: 2025-06-30.

https://clinicaltrials.gov/study/NCT04014387.

## Data Availability

The study team at the Memory & Aging Center, UCSF, will have primary access to the final trial dataset. A subset of the data will be provided to Advanced Brain Monitoring, Inc. which they will utilize to improve and further their algorithms. An application can be submitted to request de-identified data through the Memory & Aging Center, UCSF data sharing committee through the following link: https://ucsf.co1.qualtrics.com/jfe/form/SV_01EHhRABhqgPgO2.

## Abbreviations

AEs: Adverse events
AD: Alzheimer’s disease
CRF: Case report form
CNS: Central nervous system
CGI-C: Clinical Global Impression Change
CGI-ds: Clinical Global Impression Disease Severity score
CBFS: Cortical Basal Functional Rating Scale
CBS-PSP: Corticobasal syndrome PSP
DSMB: Data and Safety Monitoring Board
EEG: Electroencephalogram
EMG: Electromyogram
EOG: Electrooculography
ESS: Epworth Sleepiness Scale
FDA: Food and Drug Administration
FTD: Frontotemporal Dementia
GAD-7: General Anxiety Disorder
HIPAA: Health Insurance Portability and Accountability Act
ISI: Insomnia Severity Index
IRB: Institutional Review Board
LHA: Lateral hypothalamus
LC: Locus coeruleus
MAC: Memory and Aging Center
MAR: Missing at random
MSLT: Multiple sleep latency test
NREM: Non-REM
PHQ-9: Patient Health Questionnaire
PPA-PSP: Primary progressive aphasia
PI: Principal investigator
PHI: Private health information
PAGF-PSP: Progressive akinesia with gait freezing PSP
PSP: Progressive supranuclear palsy
PSP-QoL: PSP quality of life
REM: Rapid eye movement
PSP-RS: Richardson’s syndrome
SSS: Stanford Sleepiness Scale
SAE: Serious adverse event
TMN: Tuberomammillary nucleus
UCSF: University of California, San Francisco
WPN: Wake-promoting neurons
WIRB: Western Institutional Review Board
